# Patient information in radiation oncology: a cross-sectional pilot study using the EORTC QLQ-INFO26 module

**DOI:** 10.1186/1748-717X-4-40

**Published:** 2009-09-28

**Authors:** Johannes Adler, Yvonne Paelecke-Habermann, Patrick Jahn, Margarete Landenberger, Bernd Leplow, Dirk Vordermark

**Affiliations:** 1 Department of Radiation Oncology, Martin Luther University Halle-Wittenberg, Halle/Saale, Germany; 2 Department of Psychology, Martin Luther University Halle-Wittenberg, Halle/Saale, Germany; 3 Institute of Nursing and Health Science, Martin Luther University Halle-Wittenberg, Halle/Saale, Germany

## Abstract

**Background:**

The availability of alternative sources of information, e. g. the internet, may influence the quantity and quality of information cancer patients receive regarding their disease and treatment. The purpose of the present study was to assess perception of information in cancer patients during radiotherapy as well as media preferences and specifically the utilization of the internet.

**Methods:**

In a cross-sectional, single-centre study 94 patients currently undergoing radiotherapy were asked to complete two questionnaires. The EORTC QLQ-INFO26 module was used to assess the quality and quantity of information received by patients in the areas disease, medical tests, treatment, other services, different places of care and how to help themselves, as well as qualitative aspects as helpfulness of and satisfaction with this information. The importance of different media, in particular the internet, was investigated by a nine-item questionnaire.

**Results:**

The response rate was n = 72 patients (77%). Patients felt best informed concerning medical tests (mean ± SD score 79 ± 22, scale 0-100) followed by disease (68 ± 21). Treatment (52 ± 24) and different places of care and other services (30 ± 36 and 30 ± 30, respectively) ranked last. 37% of patients were very satisfied and 37% moderately satisfied with the amount of information received, 61% wished more information. Among eight media, brochures, television and internet were ranked as most important. 41% used the internet themselves or via friends or family, mostly for research of classic and alternative treatment options. Unavailability and the necessity of computer skills were most mentioned obstacles.

**Conclusion:**

In a single-center pilot study, radiotherapy patients indicated having received most information about medical tests and their disease. Patients very satisfied with their information had received the largest amount of information. Brochures, television and internet were the most important media. Individual patient needs should be considered in the development of novel information strategies.

## Background

Patient information in oncology has recently received increasing attention. Providing adequate information to the cancer patient can reduce anxiety and improve compliance of the patient [1]. Information disclosure adapted to the individual needs of each patient can facilitate patient-physician communication to enable informed decisions [2] and increase patient satisfaction [3]. Despite present efforts to improve cancer patient information, recent studies reported between 10 and 28% of patients not satisfied with the information [4,5]. Whereas several studies investigated the amount and content of information [6], others focused on its influence on quality of life [7].

From the physician's perspective, the information needs of an individual cancer patient may not always be easily detectable [8]. One finding in published studies was that patients wanted as much information as possible [9,10]. On the other hand, satisfaction with the information provided may be more relevant for the quality of life of an individual cancer patient than the mere quantity of information [7].

New media have been introduced for providing information to cancer patients. Written information supplied routinely during doctor consultation has been supplemented by interactive DVDs [11] and audiotapes of the consultation [12]. The internet is gaining more importance as a source of information for cancer patients [13-16].

The aim of the present study was to evaluate the perception of cancer patients undergoing radiotherapy regarding the information received on different areas of the disease, diagnosis, treatment and care and the importance of different media, especially the internet.

## Methods

We conducted a cross-sectional observational study at a single center to generate hypotheses for future larger-scale studies of patient information in cancer patients. We now included cancer patients undergoing radiotherapy at the Department of Radiation Oncology, Martin Luther University Halle-Wittenberg, Germany, in November 2008.

All patients on treatment were eligible regardless of age, tumor entity or type of radiotherapy. Signed written informed consent was obtained. Patients were excluded if they were considered by the treating physicians to be unable to participate due to very poor general condition (ECOG = 4) or severe mental impairment. The study was approved by the local ethics committee.

Standard information handed out to patients at the Dept. of Radiation Oncology at the time of the study was the information part of the informed consent form. Free brochures from the German Cancer Aid (Deutsche Krebshilfe) were available in the waiting areas. Other sources of information were not routinely recommended.

The utilized INFO26 questionnaire of the EORTC [17,18] consists of 26 items organized in four scales on information about the disease (four items), about medical tests (three items), about treatment (seven items), about other services (four items) and of four single items on information in other areas as well as four items on qualitative aspects (e. g. satisfaction with amount of information, helpfulness of information). Two of the items on information in other areas asked if the patients had received written information or information on CD (yes/no). For quantitave questions, patients were asked to indicate if they received no, little, moderate or very much information on a particular subject. The answers for multi-item scales or individual items were linearly transformed to a 0-100 scale, a high score equaling a high level of information. The INFO26 questionnaire was designed and evaluated by the EORTC Quality of Life Group following a strict procedure established for module development, as described previously [17,18].

Data on the use of the internet as a source of medical information, main topics of interest and the importance of various media were collected by a previously used nine-item-questionnaire [19]. Eight possible media (television, patient brochure, newspaper, medical books, magazines, "waiting-room chat", patient-support groups and internet) were ranked from "unimportant" (0) to "very important" (2) as sources of medical information. If a specific medium was not rated by the patient, this medium was scored as "unimportant" (0).

For each scale or item of EORTC QLQ-INFO26 and for each medium, the mean and SD scores were calculated for the overall group and subgroups.

## Results

Of the overall group of n = 94 patients, those with very poor general condition or severe mental problems (n = 19) were excluded from the analysis. Of the remaining 75 patients three declined participation and 72 completed the questionnaire. Descriptive characterization of the patient cohort is presented in Table 1.

**Table 1:** Characteristics of n = 72 tumor patients (ENT: ear, nose and throat)

		**overall cohort n = 72**	
		**n**	**%**
**diagnosis**	**ENT**	15	21
	**uterus**	3	4
	**brain**	6	8
	**rectal**	8	11
	**lung**	8	11
	**esophagus/gastric**	3	4
	**breast**	13	18
	**prostate**	6	8
	**lymphoma**	5	7
	**brain metastasis**	1	1
	**others**	4	6
			
**sex**	**male**	43	60
	**female**	29	40
			
**age**	**<30**	4	6
	**40-49**	10	14
	**50-59**	18	25
	**60-69**	19	26
	**>69**	21	29
			
**education**	**basic ("Volkschule/Hauptschule")**	28	39
	**intermediate("Mittlere Reife")**	22	31
	**high("Abitur")**	4	6
	**university degree**	8	11

**Figure 1:**
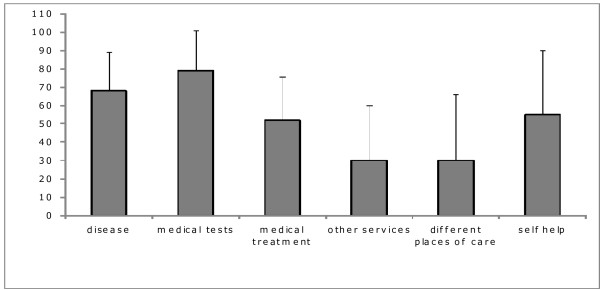
**Amount of information received by cancer patients undergoing radiotherapy on a scale from 0 to 100 (mean ± SD) concerning information about the disease, medical tests and treatment, about other services (e. g. physiotherapy), different places of care and about information to help themselves at home, as assessed by EORTC QLQ-INFO26 module (score of 100: maximum amount of information received)**.

Figure 1 depicts scales of the INFO26 questionnaire representing the quantity of information received by patients about the disease, medical tests, medical treatment, other services, different places of care and how to help themselves. High values represent a large amount of information received (maximum score 100). The mean scores range from 79 ± 22 (medical tests) to 30 ± 30 and 30 ± 36 ("other services" and "different places of care", respectively). Most information was received about the purpose, results and procedure of the medical tests, followed by information on diagnosis, spreading and possible causes of the disease and whether it is under control ("diagnosis"). Information related to medical treatment was rated forth. Less information had been received in the area "other services" (including information on patient support groups, nursing at home, rehabilitation, coping with the disease at home and psychological support) and the item "different places of care" (referring to other hospital, nursing services and nursing home).

**Figure 2:**
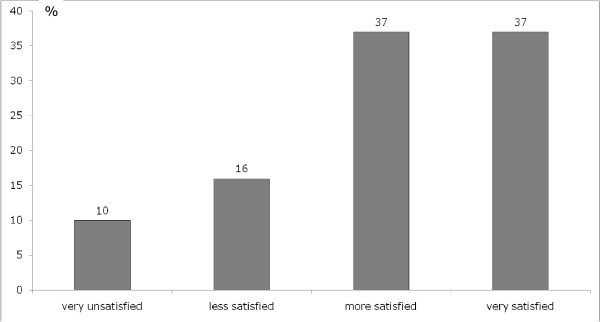
**Percentage of cancer patients undergoing radiotherapy stating that they are very unsatisfied, less, moderately or very satisfied with the amount of information received, as assessed by EORT QLQ-INFO26 module**.

Most patients were moderately to very satisfied (74%) with the amount of information about their medical issues (Figure 2). However, 61% of participants indicated that they wished more information. Topics of additional information mentioned by the patients in response to an open question for instance were further therapies, alternative treatment, nutrition and chance of cure. On the other hand, 6% of patients wanted less information. 50% of patients found the information they received as overall helpful and 6% as not helpful. Table 2 displays the differences in amount of information received in subgroups with different levels of satisfaction, showing that very satisfied patients had received more information than less satisfied patients.

**Table 2:** Amount of information (100: max. amount) by satisfaction with information

	**amount of information** **(mean ± SD score)**					
**satisfaction with amount of information**	**disease**	**medical** **tests**	**treatment**	**other services**	**different places of care**	**self-help**
**very satisfied**	83 ± 16	92 ± 19	73 ± 14	51 ± 30	58 ± 37	82 ± 26
**moderately, less satisfied and very unsatisfied**	61 ± 20	70 ± 20	41 ± 20	19 ± 23	15 ± 25	41 ± 30
**overall group**	68 ± 21	78 ± 22	53 ± 24	30 ± 30	30 ± 36	56 ± 35

**Figure 3:**
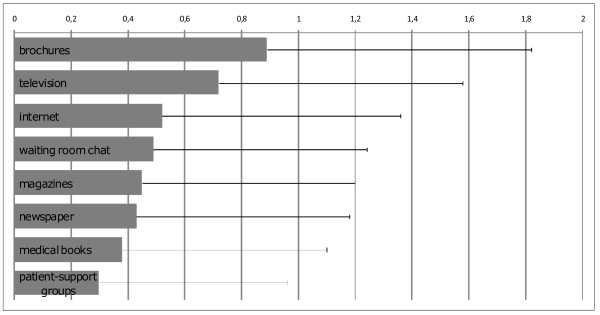
**Eight possible information media and their mean ± SD importance ranked by cancer patients undergoing radiotherapy from unimportant (0) to very important (2)**. Data are displayed as mean ± SD scores.

The importance of eight possible media (brochures, television, newspapers, magazines, internet, medical books, self-support-groups and waiting-room chat) was ranked by the cancer patients on a scale from 0 (not important) to 2 (very important) (Figure 3). The three most important media were brochures (0.89 ± 0.93), television (0.72 ± 0.86) and internet (0.52 ± 0.84). Self-support groups ranked last.

41% of patients indicated having obtained information about the disease by the help of the internet. 83% were familiar with the term 'internet' and 73% knew about the possibility of searching information on it. Main topics were classic treatment modalities (surgery, chemo- and radiation therapy) and alternative treatment options. However only 39% of internet users discussed details of information gained from the internet with their physician. The lack of sufficient computer literacy (26%) and the availability of a computer (51%) were the most frequently mentioned obstacles to using the internet for information.

**Figure 4:**
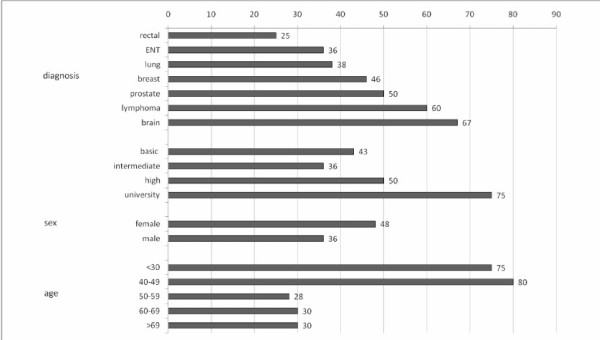
**Frequency in percent of internet use among subgroups of cancer patients undergoing radiotherapy**.

Figure 4 shows the percentage of internet use in patient subgroups by tumor entity, education, sex and age. High educational status, female sex and younger age were associated with a higher rate of internet use. Rectal and ear, nose and throat (ENT) cancer patients used the internet less frequently than brain tumor or lymphoma patients.

## Discussion

In the present cross-sectional pilot study, patients considered the received information overall helpful, even if there were still some topics partly unadressed. This concerns information about rehabilitation, self-support-groups, rehabilitation and psychological services and diverse outpatient nursing services. Comparison of satisfaction with amount of information suggested that the two are associated and that there may be a threshold level of information quantity that should be achieved in a clinical setting.

There appears to be no need in reaching the maximum quantity of information, as patients already felt very satisfied at mean scores of 82 for disease, 92 for medical tests, 73 for treatment, 51 for other services, 58 for different place of care and 82 for help at home (Table 1).

It remains unclear whether the less satisfied patients did not receive sufficient information or the patients lost information, due limited capacity and the enormous amout of information as Sadler et al showed previously [20].

We could not detect one specific medium that is particularly suitable for filling gaps of information. The internet is increasingly accepted as a source of medical information [13-16] and is now a major source of patient information (Table 3). However, in the majority of patients the information received via the internet is not communicated to the physician. Matthews et al. [21] give cause for serious concern, because online medical information is unregulated and of questionable accuracy.

**Table 3:** Importance of media for cancer patients as ranked in published studies

	**Vordermark** **2000 **[19]	**Schäfer** **2002 **[22]	**Peterson** **2003 **[15]	**Basch** **2004 **[16]	**Adler** **[this study]**
**setting**	**radiation oncology center**	**radiation oncology center**	**lung cancer center**	**cancer center**	**radiation oncology center**
**internet**	8	5	3	2	3
**doctor**	-	1	1+2	-	-
**self-support group**	7	-	-	-	8
**TV**	1	3	7	-	2
**newspaper**	3	3	-	1	6
**brochures**	2	-	-	1	1
**medical book**	4	2	5	1	7
**Waiting-room chat**	6	-	-	-	4
**telephone**	-	-	-	3	-
**family**	-	-	4	-	-
**radio**	-	5	-	-	-
**magazine**	5	3	6	1	5

Considering the average age of cancer patients (69 years at first diagnosis in Germany), the applicability of the internet to provide information to cancer patients is currently still limited by the necessity of computer skills. In less educated and older groups of patients, who showed the lowest rates of internet use in this study, alternative media should be considered.

The quantity and quality of information offered at cancer centers and the potential of new information strategies to influence the satisfaction of patients with the received information as well as their anxiety and compliance should be investigated in further studies. Some new media have been introduced as part of trials, but are not yet routinely available at German radiotherapy centers. However it remains unclear which information models are capable of fulfilling the needs of various types of patients and relatives. Passalacqua et al. [5] hypothesized that a structured modality of providing information reduces psychological distress and performed a two-arm, cluster-randomized trial implementing a point of information and support in oncology wards. It was realized by a library for cancer patients managed by a specially trained oncology nurse. End points were reduction of anxiety and depression of cancer patients. This approach did not lead to a significant reduction in anxiety and depression. Compliance was low, in more than 50% of the participating centers the library did not follow the protocol, mostly because of conflicts of staff.

Several methodological aspects of our study deserve further discussion. This trial assessed in a cross-sectional design the perception of information regarding the information received on different areas of the disease, diagnosis, treatment and care and the importance of different media, especially the internet in the distinct group of cancer patients undergoing radiotherapy. We used with EORTC QLQ-INFO26 a tested and multi-language questionnaire, and collected comparable and generalisable data. However, several limitations of this trial should be noted. First of all this was a single-centre study and the results are only representative for the region of Southern Saxony-Anhalt, a region about 1.2 million inhabitants. The perception of information received may vary during a course of radiotherapy and future studies should aim to assess patients at defined timepoints before, during or after treatment.

Future trials should use information disclosure adapted to the patients' characteristics and needs, not neglecting that there are also patients wanting less information. For instance, different media should be employed to reach a head and neck cancer patient than to address a brain tumor patient. It appears promising to integrate individual patient counseling and structured patient information provided via suitable media into comprehensive patient information strategies. In larger studies, instruments like INFO26 can help to identify the information needs of subgroups with specific tumor types.

## Conclusion

In a single-center pilot study, radiotherapy patients indicated having received most information about medical tests and their disease, less about the medical treatment or other areas. Patients very satisfied with their information had received the largest amount of information. Brochures, television and internet were the three most important media. Information needs of patients differ between tumor entities which should be considered in the development of novel information strategies.

## Competing interests

The authors declare that they have no competing interests.

## Authors' contributions

JA participated in the study design, collected the data, performed the data analysis and drafted the manuscript. YPH participated in the study design, performed the data analysis and helped to draft the manuscript. PJ participated in the study design, performed the data analysis and helped to draft the manuscript. ML participated in the study design and helped to draft the manuscript. BL participated in the study design and helped to draft the manuscript. DV participated in the study design, collected the data, performed the data analysis and helped to draft the manuscript. All authors read and approved the final manuscript.

## References

[B1] Butow PN, Kazemi JN, Beeney NJ, Griffin AM, Dun SM, Tattersall MH (1996). When the diagnosis is cancer: patient communication experiences and preferences. Cancer.

[B2] Arraras JL, Illaramendi JJ, Valerdi JJ, Wright S (1995). Truth telling to the patient in advanced care: family information filtering and prospects for change. Psychooncology.

[B3] Degner LF, Davison BJ, Sloan JA, Mueller B (1998). Development of a scale to measure information needs in cancer care. J Nurs Meas.

[B4] Montomery C, Lydon A, Lloyd K (1999). Psychological Distress among cancer patients and informed consent. J Psychosom Res.

[B5] Passalacqua R (2009). Prospective, multicenter, randomized trial of a new organizational modality for providing information and support to cancer patients. J clinical Oncol.

[B6] Butow PN, Kazemi JN, Beeney NJ, Griffin AM, Dun SM, Tattersall MH, Boyer MJ (1997). The dynamics of change: cancer patient's preferences for information involvement and support. Ann Oncol.

[B7] Annunziata MA, Foladore S, Margi MD, Crivellari D, Feltrin A, Bidoli E, Veronesi A (1998). Does information level of cancer patients correlate with quality of life? A prospective study. Tumori.

[B8] Blanchard CG, Labreque MS, Ruckdeschel JC, Blanchard EB (1988). Information and decision-making preferences of hospitalized cancer patients. Soc Sci Med.

[B9] Gysels M, Higginson IJ (2007). Interactive technologies and videotapes for patient education in cancer care: systematic review and meta-analysis of randomized trials. Support Care Cancer.

[B10] Fallowfield l, Lord S, Lewis S (1995). No news is not good news: information preferences of patients with cancer. Psychooncology.

[B11] Strevel EL, Newman C, Pond GR, MacLean M, Siu LL (2007). The impact of educational DVD on cancer patients considering participation in a phase I clinical trial. Support Care Cancer.

[B12] Brucera E, Pituskin E, Calder K, Neumann CM, Hanson J (1999). The addition of an audiocassette recording of a consultation to written recommendations for patients with advanced caner: a randomized trial. Cancer.

[B13] Van de Poll-Franse L, van Ellenbergen MC (2007). Internet use by cancer survivors: current use and future wishes. Support Care Cancer.

[B14] Chen X, Siu LL (2001). Impact of the media and the internet on oncology: survey of cancer patients and oncologists in Canada. J Clin Oncol.

[B15] Peterson MW, Fretz PC (2003). Patient Use of the Internet for Information in a Lung Cancer Clinic. Chest.

[B16] Basch EM, Thaler HT, Shi W, Yakren S, Schrag D (2004). Use of information resources by patients with cancer and their companions. American Cancer Society.

[B17] Arraras JI (2007). EORTC QLQ-INFO 26: A questionnaire to assess information given to cancer patients a preliminary analysis in eight countries. Psycho-Oncology.

[B18] Arraras JI, Wrigth S, Greimel E, Holzner B, Kuljanic-Vlasic K, Velikova G, Eisemann M, Visser A (2004). Development of a questionnaire to evaluate the information needs of cancer patients: the EORTC questionnaire. Patient education and Counseling.

[B19] Vordermark D, Kolbl O, Flentje M (2000). The internet as a source of medical information: investigation in a mixed cohort of radiotherapy patients. Strahlenther Onkol.

[B20] Sadler GR, Oberle-Edwards L, Farooqi A, Hryniuk WM (2000). Oral sequelae of chemotherapy: an important teaching opportunity for oncology health care providers and their patients. Support Care Cancer.

[B21] Matthews SC, Camacho A, Mills PJ, Dimsdale JE (2003). The internet for medical information about cancer. Help of hindrance?. Psychosomatics.

[B22] Schäfer C, Dietl B, Putnik K, Altmann D, Marienhagen J, Herbst M (2002). Patient information in Radiooncology. Strahlenther Onkol.

